# Supplementary material for: Molecular Epidemiology of Carbapenem-Resistant Klebsiella aerogenes in Japan
Source: Int J Mol Sci. 2024 Apr 19;25(8):4494. doi: 10.3390/ijms25084494 (PMC11049973; doi:10.3390/ijms25084494)
Supplement: Supplementary file 1 [file ijms-25-04494-s001.zip › Table S1.pdf]

Table S1. Drug sensitivity test results for carbapenemase-producing *Klebsiella aerogenes*.

Bacterial strains	TUT0013	TUT0015	TUT0023	TUT0032	TUT0037	TUT0049
Antimicrobial drugs	Antibiotic Minimum Inhibitory Concentration (μg/mL)					
Piperacillin	≥128	−	≥128	≥128	≥128	≥128
Tazobactam/piperacillin	=32	≤16	≤16	≤16	−	≥128
Cefmetazole	≥64	−	≥64	≥64	≥64	≥64
Ceftriaxone	≥4	≥4	≥4	≥4	−	≥4
Cefepime	≤2	−	≥32	≥32	≥32	≥32
Meropenem	≥4	≥4	≥4	≥4	−	≥4
Imipenem/Cilastatin	=2	−	−	−	≥4	−
Azactam	≤4	−	≥16	≥16	≤4	≤4
Gentamicin	≤2	−	≤2	≤2	≤2	≤2
Amikacin	≤4	−	−	−	≤4	≤4
Fosmicin	≤4	=16	=16	≥32	≤4	≤4
Levofloxacin	≤0.5	=1	≤0.5	≤0.5	≤0.5	≤0.5
Trimethoprim/Sulfamethoxazole	≤38	−	≥76	≤38	≤38	≤38

*Each number represents Breakpoint and MIC values. A dash indicates not measured.
