# Supplementary material for: Molecular Epidemiology of Carbapenem-Resistant Klebsiella aerogenes in Japan
Source: Int J Mol Sci. 2024 Apr 19;25(8):4494. doi: 10.3390/ijms25084494 (PMC11049973; doi:10.3390/ijms25084494)
Supplement: Supplementary file 1 [file ijms-25-04494-s001.zip › Table S2.pdf]

Table S2. Antimicrobial resistance genes and mobile genetic genes in this study.

Abbreviation	Name or function of each gene
aadA2	Aminoglycoside adenyltransferase type 2
aac(6')-Ib (aacA4)	Aminoglycoside 6'-N-acetyltransferase type Ib
aph(3')-XV	Aminoglycoside phosphotransferase (3')-XV
bla$_{CTX-M-2}$	CTX-M-2 type β-lactamase gene
bla$_{OXA-101}$	Oxacillinase-101 gene
bla$_{SHV-190}$	SHV-190 extended Spectrum beta(β)-Lactamase gene
ble	Bleomycin resistance gene
dfrA14	Quinolone resistance gene
dsbD	Disulfide bond protein family D gene
fosA7	Fosfomycin resistance gene
IS5	Insertion Sequence element, IS5 family
IS3000	Insertion Sequence element, Tn3 family
ISAba125	Insertion Sequence element, IS30 family
ISKox3	Insertion Sequence element, ICL3 family
ISVsa10	Insertion Sequence element, IS91 family
ORF	A sequence of DNA that has the potential to be translated into a protein
oqxA	Quinolone resistance gene
qnrS1	Quinolone resistance gene
sul1	Sulfonamide resistance gene 1
tet(A)	Tetracycline resistance gene
Tn2	Transposase, Tn3 family
Tn5403	Transposase, Tn3 family
trpF	N-(5'-phosphoribosyl)anthranilate isomerase
