# Supplementary material for: Molecular Epidemiology of Carbapenem-Resistant Klebsiella aerogenes in Japan
Source: Int J Mol Sci. 2024 Apr 19;25(8):4494. doi: 10.3390/ijms25084494 (PMC11049973; doi:10.3390/ijms25084494)
Supplement: Supplementary file 1 [file ijms-25-04494-s001.zip › Table S3.pdf]

Table S3. Domestic and international representative integrons harboring *bla*$_{IMP-1}$ and *bla*$_{IMP-6}$ referenced in our Study.

Carbapenemase	Species	Integron structure including gene cassettes	Integron no.	Country	Genbank accession no.
IMP-1	*Escherichia coli*	intl1-aacA1-*bla*$_{IMP-1}$-Emr-qacE-sul1	In798	Japan	LC720959
	Klebsiella pneumoniae	intl1-aacA4-*bla*$_{IMP-1}$-aadA2-Tnp-qacE-sul1	In1311	Japan	LC169566
	Citrobacter freundii	intl1-*bla*$_{IMP-1}$-aacA31-*bla*$_{OXA142}$-qacE-sul1	In1312	Brazil	LC169567
	Klebsiella pneumoniae	intl1-*bla*$_{IMP-1}$-ORF-aacA4-aadA6-Tnp	In1223	China	KT345947
	Enterobacter hormaechei	intl1-*bla*$_{IMP-1}$-aacA4-cr-sul1-ORF	In1426	Japan	AP018351
IMP-6	*Klebsiella pneumoniae*	intI1-aacA4-*bla*$_{IMP-6}$-aadA2-Tnp-qacE-sul1	In1321	Japan	LC169589
	Klebsiella pneumoniae	intI1-aacA4-*bla*$_{IMP-6}$-aadA2-Tnp-qacE-sul1	In1547	Japan	AP019404
	Escherichia coli	intI1-aacA4-*bla*$_{IMP-6}$-aadA2-qacE-sul1	In1690	Japan	LC720960
	Klebsiella pneumoniae	s. a	In1690	Japan	AP019402
	Klebsiella aerogenes	s. a	In1690	Japan	AP019272

EMR stands for a efflux-mediated small multidrug resistance (SMR) transporter. The transposases (Tnp) of In1321 and In1547 differ from each other, and the alleles of adenine and thymine at position 368 of the aacA4 gene also vary. To maintain consistency with original citations, what were previously named aac(6′)-Ib are here referred to as aacA4. The three integrons in In1690 are identical, thus abbreviated as "Same as above" (s.a.).
