# Supplementary material for: Molecular Epidemiology of Carbapenem-Resistant Klebsiella aerogenes in Japan
Source: Int J Mol Sci. 2024 Apr 19;25(8):4494. doi: 10.3390/ijms25084494 (PMC11049973; doi:10.3390/ijms25084494)
Supplement: Supplementary file 1 [file ijms-25-04494-s001.zip › Table S4.pdf]

Table S4. The scale and role of the targeted facility in this study.

Number of Hospital Beds	Clinic	Acute care	Rehabilitative care	Long-term care
0	2(2)	—	—	—
1-99	—	9(9)	2(2)	4(4)
100-199	—	15(18)	2(2)	3(3)
200-299	—	7(8)	0	1(1)
300-399	—	5(9)	0	2(2)
400-499	—	2(4)	0	0
500-	—	3(3)	0	0
Total	2(2)	41(51)	4(4)	10(10)

* Numbers in parentheses indicate the number of CRE isolates.

The functional classification of hospitals is based solely on the function of the medical care provided. Hospitals with some convalescent and chronic care units in acute care hospitals were classified as acute care hospitals if the medical care provided was primarily acute care. Hospitals with primarily convalescent beds were defined as rehabilitation hospitals, and hospitals providing exclusively chronic care were defined as long-term care hospitals based on publicly available information about each hospital's beds. Although carbapenem-resistant *Klebsiella aerogenes* was most frequently reported in acute care hospitals with 100-199 beds, a chi-squared test for function (acute care, rehabilitation care, long-term care) and number of beds in facilities with beds did not detect a statistically significant difference (P = 0.0845).
